# Engineering of long-acting human growth hormone-Fc fusion proteins: Effects of valency, fusion position, and linker design on pharmacokinetics and efficacy

**DOI:** 10.1371/journal.pone.0323791

**Published:** 2025-05-15

**Authors:** Taekyeol Lee, Dongsop Lee, Eunee Jung, Mikyung Son, Kyohwan Koo, Jaehoon Choi

**Affiliations:** 1 Biologics Research Laboratories of R&D center, Dong-A Socio Holdings, Yong-In, Korea; 2 Department of Life Science, Sogang University, Seoul, Korea; Council for Scientific and Industrial Research, SOUTH AFRICA

## Abstract

Fc fusion proteins, formed by fusing an active protein to the Fc region of immunoglobulin G, are a validated strategy for extending the half-life of therapeutic proteins. Human growth hormone (hGH) Fc fusion proteins exhibit longer circulation half-lives than hGH, reducing injection frequency and improving convenience for hGH replacement therapy. Most approved Fc fusion proteins involve directly attaching the active protein to the hinge region of IgG Fc; however, few reports have described the effects of structural variations on these characteristics in detail. We analyzed pharmacokinetic and pharmacodynamic properties of various hGH-Fc fusion constructs differing in linker type, hGH valency, and fusion position to investigate the structure-function relationships of these proteins in cell-based assays and animal models, including normal and hypophysectomized rats. Monovalent hGH-Fc fusion variants and those with hGH fused to the C-terminal of IgG Fc exhibited higher *in vitro* and *in vivo* activity than bivalent hGH-Fc. However, these variants also exhibited accelerated clearance in rat pharmacokinetic experiments. The linker connecting the hGH moiety to the Fc domain significantly influenced *in vitro* activity and pharmacokinetics. Constructs with a rigid alpha-helical A(EAAAK)_5_A linker showed greater *in vitro* activity than those with a flexible (GGGGS)_3_ linker but exhibited accelerated clearance in rats. To a lesser extent, linker length influenced activity and pharmacokinetics. Bivalent hGH-Fc constructs with shorter linkers (0–1 GGGGS repeats) exhibited higher *in vivo* exposure (AUC) but lower *in vitro* activity than those with longer linkers (2–3 repeats). *In vitro* activity did not correlate linearly with linker length, as constructs with no linker (n = 0) showed reduced activity, while no consistent trend was observed for n = 1–3. These findings provide valuable insights into the design of hGH-Fc fusion proteins, offering a framework for systematically improving their potency and longevity and supporting the development of long-acting hGH therapies.

## Introduction

Growth hormone deficiency (GHD) is a rare endocrine disorder caused by insufficient secretion of somatropin produced by the pituitary gland. GHD has an estimated prevalence of 1 in every 4,000–10,000 children [1,2]. This deficiency results in inadequate levels of circulating insulin-like growth factor 1, manifesting primarily as reduced linear growth during childhood and short stature in adulthood. Additionally, GHD is associated with less apparent metabolic and phenotypic deficits and psychosocial consequences [3,4]. Without treatment, children with GHD experience persistent growth attenuation, leading to significantly reduced adult height and often delayed puberty [5].

Growth hormone replacement therapy using recombinant human growth hormone (rhGH) is a safe and effective treatment for GHD. However, it requires daily subcutaneous injections over several years, which can lead to decreased compliance and suboptimal treatment outcomes [6,7]. The inconvenience of daily injections also presents challenges for uptake in other conditions, such as adult GHD, idiopathic short stature, and Turner syndrome. To address this aspect, numerous long-acting hGH products have been developed to reduce injection frequency from daily to weekly or less. Approaches studied in clinical trials include PEGylated hGH, hGH fused to albumin, hGH fused to carboxy-terminal peptides, and fusion to long unstructured hydrophilic amino acid sequences known as XTEN [8–10]. Recently, a carboxy-terminal peptides-fused hGH product was introduced to the market under the brand name Ngenla™. This hGH analog provided once weekly is specifically approved for pediatric patients aged 3 years and above with growth failure due to insufficient endogenous growth hormone secretion [11].

Fc fusion is a well-established method to extend the half-life of therapeutic proteins, with eleven such proteins approved by the FDA [8]. The fusion of therapeutically active proteins or peptides to the Fc region of immunoglobulin G (IgG) increases their half-life by increasing molecular weight, reducing renal elimination, and enabling binding to the neonatal Fc receptor (FcRn). The FcRn recycling mechanism enables IgG and albumin to have long half-lives [12–14]. Additionally, because IgG Fc is naturally homodimeric, most Fc fusion proteins are constructed as bivalent proteins, with an active protein moiety fused to each chain of the Fc dimer. Notable exceptions include the Factor IX Fc fusion protein Alprolix® (eftrenonacog-alfa) and the Factor IX Fc fusion protein Eloctate® (efmoroctocog-alfa), which are monovalent with respect to their anti-hemophilic factor moieties [15,16].

Most approved Fc fusion proteins rely on direct fusion of the active protein to the hinge region of IgG Fc; however, limited information exists on the role of linker incorporation in this context, and the effects of structural variations remain unclear. Therefore, in this study, various design elements, including linker type, hGH valency, and fusion position, were systematically evaluated to elucidate their effects on the pharmacokinetic (PK) and pharmacodynamic (PD) properties of hGH-Fc fusion proteins. We aimed to develop a long-acting hGH analog offering a more convenient treatment schedule than the current daily regimen. We hypothesize that incorporating specific design elements in hGH-Fc fusion proteins could optimize their PK and PD profiles, resulting in the formation of a long-acting hGH analog that improves treatment convenience and efficacy.

## Materials and methods

### hGH-Fc fusion constructs

**Fig 1:**
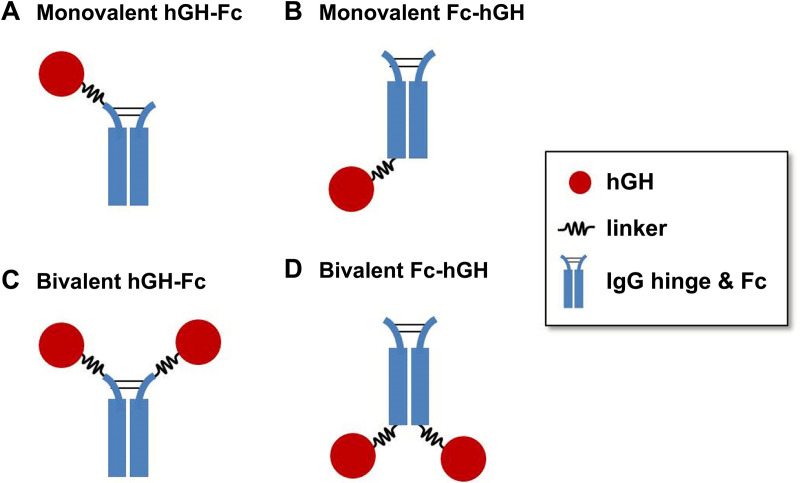
Schematic structure of human growth hormone Fc fusion proteins. **(A)** Human growth hormone (hGH) fused to the hinge region of one Fc chain (monovalent hGH-Fc). **(B)** hGH fused to the C-terminal end of one Fc chain (monovalent Fc-hGH). **(C)** hGH fused to the hinge regions of both Fc chains (bivalent hGH-Fc). **(D)** hGH fused to the C-terminal ends of both Fc chains (bivalent Fc-hGH). Table 1 shows the various fusion proteins constructed in each category.

hGH-Fc fusion proteins were constructed by genetically fusing hGH to the N-terminal hinge region or C-terminal end of one or both human IgG4 Fc region chains. The categories of hGH-Fc fusion proteins based on hGH position and valency are shown in Fig 1A–D.

**Table 1:** Construction of human growth hormone Fc fusion proteins.

Category	Construct name	M.W. (kDa)	Linker sequence
Monovalent hGH-Fc	Mono-hGH-(FL3)-Fc	73.8	GGGGSGGGGSGGGGS
	Mono-hGH-(FL2)-Fc	73.5	GGGGSGGGGS
	Mono-hGH-(FL1)-Fc	73.2	GGGGS
	Mono-hGH-(NL)-Fc	72.9	No linker
	Mono-hGH-(RL)-Fc	74.9	AEAAAKEAAAKEAAAKEAAAKEAAAKA
	Mono-hGH-(GL)-Fc	74.2	GGGNGTGGGNGTGGGNGT
Monovalent Fc-hGH	Fc-(FL3)-mono-hGH	73.8	GGGGSGGGGSGGGGS
	Fc-(RL)-mono-hGH	74.9	AEAAAKEAAAKEAAAKEAAAKEAAAKA
Bivalent hGH-Fc	Di-hGH-(FL3)-Fc	96.9	GGGGSGGGGSGGGGS
	Di-hGH-(FL2)-Fc	96.3	GGGGSGGGGS
	Di-hGH-(FL1)-Fc	95.6	GGGGS
	Di-hGH-(NL)-Fc	95.0	No linker
	Di-hGH-(RL)-Fc	99.1	AEAAAKEAAAKEAAAKEAAAKEAAAKA
	Di-hGH-(GL)-Fc	97.7	GGGNGTGGGNGTGGGNGT
Bivalent Fc-hGH	Fc-(FL3)-di-hGH	96.9	GGGGSGGGGSGGGGS

The abbreviations in parentheses indicate whether a flexible linker (“FL”), rigid linker (“RL”), linker with glycosylation sites (“GL”), or no linker (“NL”) was used to connect the hGH moiety and the Fc domain. Abbreviations: hGH, human growth hormone

A (GGGGS)_n_ linker [17,18] and an alpha-helical A(EAAAK)_5_A linker [19,20] were used to represent flexible and rigid linkers, respectively, to investigate the effect of linker characteristics on pharmacokinetics and pharmacodynamics. hGH-Fc fusion proteins with glycosylated linkers were constructed by introducing N-glycosylation sites (Asn-Gly-Thr) at three different locations in the flexible linker. Linker glycosylation was confirmed using liquid chromatography-mass spectrometry, which showed that N-glycosylation site occupancy was highest at the hGH-distal glycosylation site (S1 A–D Fig, S1 Table). cDNAs encoding hGH-Fc fusion proteins were synthesized using the GeneArt gene synthesis service (Thermo Fisher Scientific, Waltham, MA, USA). Codon optimization of cDNAs was performed optionally to enhance protein expression in the animal cell expression system.

### Expression and purification of hGH-Fc fusion proteins

Codon-optimized cDNA sequences were cloned into the Freedom^TM^ pCHO1.0 vector (Thermo Fisher Scientific). The plasmid vectors were amplified in *Escherichia coli* DH5α (RBC Bioscience, New Taipei City, Taiwan) and purified using a QIAprep® Spin Miniprep Kit (Qiagen, Hilden, Germany). hGH-Fc fusion proteins for *in vitro* and *in vivo* studies were produced by transfection and transient expression of the plasmid vectors in Chinese hamster ovary (CHO) cells using the ExpiCHO^TM^ expression system (Thermo Fisher Scientific) according to the manufacturer’s instructions. To promote heterodimerization, knobs-into-holes mutations were introduced into the hGH-Fc chains (T366W, T366S, L368A) and Fc chains (Y407V), as described previously [21].

After transfection and fed-batch flask culture for 14 days, culture supernatants were harvested, centrifuged at 218 × *g* for 5 min to remove cell debris, and filtered through 0.2-µm bottle top filters. Bivalent hGH-Fc fusion proteins were purified using a single step of protein A affinity chromatography. Briefly, clarified supernatants were loaded onto a column packed with ProSep® Ultra Plus resin (Merck Millipore, Burlington, MA, USA) pre-equilibrated with the equilibration buffer (20 mM potassium phosphate, pH 6.8). After washing the column with three bed volumes of equilibration buffer, the bound protein was eluted with 100 mM glycine (pH 3.0) and neutralized with 1 M Tris-HCl (pH 9.0) to a final pH of 7.0.

Monovalent hGH-Fc fusion proteins were first purified using hGH affinity chromatography. Clarified supernatants were loaded onto a column packed with CaptureSelect^TM^ Human Growth Hormone Affinity resin (Thermo Fisher Scientific) equilibrated with phosphate-buffered saline (PBS). After washing with two bed volumes of equilibration buffer, the bound protein was eluted with 20 mM citric acid and applied as a linear gradient from 0 to 100% over four bed volumes. Purified proteins were neutralized with 1 M Tris-HCl (pH 9.0) to a final pH of 7.0.

For constructs with low purity, further purification was achieved using hydrophobic interaction chromatography or anion exchange chromatography, depending on the construct. For hydrophobic interaction chromatography, 4.5 M sodium chloride was added to the product pool from the previous step to a final concentration of 3 M. The adjusted pool was loaded onto a HiTrap^TM^ Phenyl HP column (GE Healthcare, Chicago, IL, USA) equilibrated with PBS containing 3 M sodium chloride. After washing with three bed volumes of equilibration buffer, the bound protein was eluted with PBS and applied as a linear gradient from 0 to 100% over four bed volumes. For anion exchange chromatography, the pool from the previous step was loaded onto a column packed with Fractogel® TMAE(s) (EMD Millipore, Burlington, MA, USA) and equilibrated with 20 mM Tris-HCl buffer (pH 7.4). After washing with three bed volumes of equilibration buffer, the bound protein was eluted with 20 mM Tris-HCl and 500 mM sodium chloride (pH 7.4) and applied as a linear gradient from 0 to 100% over four bed volumes. Purified hGH-Fc fusion proteins were dialyzed against PBS, sterilized by filtration through 0.22 µm syringe filters, and stored at -20°C.

Expression of fusion proteins was confirmed by western blot analysis using an anti-human IgG (Fc-specific)−alkaline phosphatase antibody produced in goats (Sigma-Aldrich, St. Louis, MO, USA) after blotting the proteins separated by sodium dodecyl sulfate-polyacrylamide gel electrophoresis (SDS-PAGE) onto low-fluorescence polyvinylidene fluoride transfer membranes (Thermo Fisher Scientific). Purified proteins were also analyzed using SDS-PAGE under non-reducing and reducing conditions. SDS-PAGE was performed using NuPAGE™ 4–12% Bis-Tris gel (Thermo Fisher Scientific) according to the manufacturer’s instructions. Proteins were separated in NuPAGE MES SDS running buffer (Thermo Fisher Scientific) and visualized with PhastGel® Blue R (Sigma-Aldrich).

### *In vitro* activity in Nb2 cell proliferation assay

The *in vitro* activities of hGH-Fc fusion proteins were evaluated using a proliferation assay with the pre-T rat lymphoma Nb2–11 cell line (Sigma-Aldrich), based on a modified method previously described [22]. The Nb2 cell line responds to hGH through a truncated prolactin receptor and is not strictly specific to hGH. However, the assay was deemed appropriate for measuring hGH analog activity because prolactin and growth hormone receptors share high structural similarity and nearly identical structural epitopes on hGH [23]. Nb2 cells were routinely cultured in Fisher’s medium (Thermo Fisher Scientific) supplemented with 10% horse serum (Gibco, Waltham, MA, USA), 10% fetal bovine serum (Gibco), 0.05 mM 2-mercaptoethanol (Gibco), and antibiotics (50 U/mL penicillin; Sigma-Aldrich, 50 µg/mL streptomycin; Sigma-Aldrich) at 37°C in a humidified incubator with 5% CO_2_.

Approximately 60 h before the activity assay, Nb2 cells were transferred to fetal bovine serum-free Fisher’s medium with 1% horse serum, 0.05 mM 2-mercaptoethanol, and antibiotics to obtain stationary cultures with reduced replication rates. After incubation, cells were collected by centrifugation (3 min at 218 × *g*) and resuspended in assay medium (Fisher’s medium supplemented with 10% horse serum, 0.05 mM 2-mercaptoethanol, and antibiotics) at a density of 2 × 10^5^ cells/mL. The cell suspension was dispensed into 96-well microplates (200 µL per well) and incubated for 3 days with 25 µL of serially diluted samples. Cell proliferation was quantified by adding 0.1 volume of Alamar Blue® reagent (My BioSource, San Diego, CA, USA) and measuring fluorescence emission at 590 nm (excitation wavelength 590 nm) after approximately 10 h of incubation. Raw RFU values and EC50 calculations for all constructs are available in S1 raw data*.*

### *In vivo* study

#### Animals.

The animal experiments were approved by the Institutional Animal Care and Use Committee of Dong-A ST (Yogin, Korea) (Approval Number: No. I-1607198, No. I-1609235, No. I-1611287, No. I-1703067, No. I-1607194, No. I-176135, No. I-1708171) and conducted in accordance with the recommendations outlined in the Guide for the Care and Use of Laboratory Animals of the National Institutes of Health. All animal procedures were carefully planned to minimize any potential pain or distress. Seven-week-old, male Sprague–Dawley (SD) rats weighing approximately 250 g were obtained from Samtako Bio Korea (Osan, Korea), while 5-week-old hypophysectomized male SD rats weighing approximately 100 g were sourced from Japan SLC Inc. (Shizuoka, Japan). Upon arrival, all animals were acclimated to the testing facilities for at least one week before the start of any procedures. All animals were maintained on a 12-h light/dark cycle in a controlled environment with a room temperature of 22 ± 2°C and humidity levels of 40–60%. Rats were provided with ad libitum access to a standard rodent chow diet and filtered water throughout the study period. All surgeries were performed under sodium pentobarbital anesthesia, and all efforts were made to minimize animal suffering, including analgesic administration post-operatively when signs of pain were observed. Pain was assessed using a grimace scale and activity levels were monitored. Animals exhibiting signs of persistent pain or distress despite analgesic treatment were immediately euthanized. At the end of the study, animals were euthanized by CO_2_ inhalation followed by cervical dislocation to ensure death, a method consistent with the recommendations of the American Veterinary Medical Association (AVMA) Guidelines for the Euthanasia of Animals. CO_2_ was administered at a gradual displacement rate (10–30% volume/min) to minimize distress. Animals were closely monitored during CO_2_ exposure to confirm unconsciousness before cervical dislocation

#### Pharmacokinetic evaluation.

The PK profiles of hGH-Fc fusion proteins were evaluated in male SD rats (five per group) administered single subcutaneous doses of 1 mg/kg of hGH-Fc fusion protein based on individual body weights measured 1 day prior to dosing. Blood samples were collected before dosing and at predetermined time points, allowed to coagulate at room temperature (22 ± 2°C) for 30 min, and centrifuged at 1,500 × *g* for 15 min. Serum samples were stored at temperatures below -60°C until analysis. Serum concentrations of hGH-Fc fusion proteins were measured using a commercial hGH ELISA kit (Quantikine® Human Growth Hormone Immunoassay, R&D Systems, Minneapolis, MN, USA). Purified hGH-Fc fusion proteins were used to generate construct-specific standard curves. PK parameters, including maximum serum concentration (C_max_), time to reach C_max_ (T_max_), area under the serum concentration-time curve (AUC), and half-life (t_1/2_), were determined using non-compartmental analysis with WinNonlin (Pharsight, version 6.2). Concentrations below the assay quantification limit were set to zero for parameter estimation. For most constructs, terminal half-lives were calculated using data from days 3–7, which exhibited a consistent elimination rate. For rapidly clearing proteins, terminal half-lives were determined using three or more time points that provided the best fit in the WinNonlin program. Complete datasets including individual rat serum concentration-time profiles are provided in S1 raw data*.*

#### Hypophysectomized rat studies.

The efficacy of various hGH-Fc fusion proteins was assessed in hypophysectomized rats by evaluating body weight gain and tibial growth plate width following treatment. Before the study, animals were acclimated to the testing facilities for 1 week and monitored for body weight changes. Rats showing abnormal weight changes (< 5%) during the acclimation period were excluded from the study to ensure the accuracy of the results. On the day before dosing, animals were randomized into treatment groups based on body weight to ensure balanced average body weights across groups. Treatment groups received either daily subcutaneous injections of a commercial rhGH product (15 µg/head; Growtropin®, Dong-A ST, Korea) for 14 days (days 0–13) or two weekly subcutaneous injections (days 0 and 7) of the hGH-Fc fusion protein. For hGH-Fc constructs, doses were adjusted to deliver the hGH-equivalent content of 15 µg/head/day (or 7.5 µg/head/day in some groups) to account for differences in molecular weight between constructs.

Body weights were recorded daily for 14 days after the first injection. On day 14, animals were euthanized, and tibias were harvested for histological analysis. The right tibias were fixed in 10% neutral buffered formalin, decalcified, embedded in paraffin, and sectioned into 8-µm slices using a microtome. The sections were stained with toluidine blue for histological examination of the epiphyseal growth plate. Tibial growth plate width was defined as the distance from the undifferentiated layer to the hypertrophic layer and measured using ImageJ software (National Institutes of Health, Bethesda, MD, USA). For each sample, the average of three measurements was used to determine the growth plate width. Daily body weight measurements and tibial growth plate width data from hypophysectomized rats are compiled in S1 raw data

### Statistical analysis

All statistical analyses were performed using SigmaStat Software v4.0 (SPSS Inc., Chicago, IL, USA). Data are presented as mean ± standard deviation (SD) unless otherwise specified. Differences in pharmacokinetic parameters (C_max_, T_max_, AUC, and t_1/2_), body weight gain, and tibial growth plate widths (between the hGH-Fc fusion protein constructs and control groups) were evaluated. For pharmacokinetic studies and tibial growth plate width measurements, a one-way analysis of variance (ANOVA) was used to determine statistical significance. Repeated measures ANOVA was applied to assess differences in body weight gain over time in hypophysectomized rats. Post-hoc comparisons were performed using Tukey’s test for multiple comparisons. A p-value < 0.05 was considered significant for all analyses.

## Results

### hGH-Fc fusion protein expression and purification

**Fig 2:**
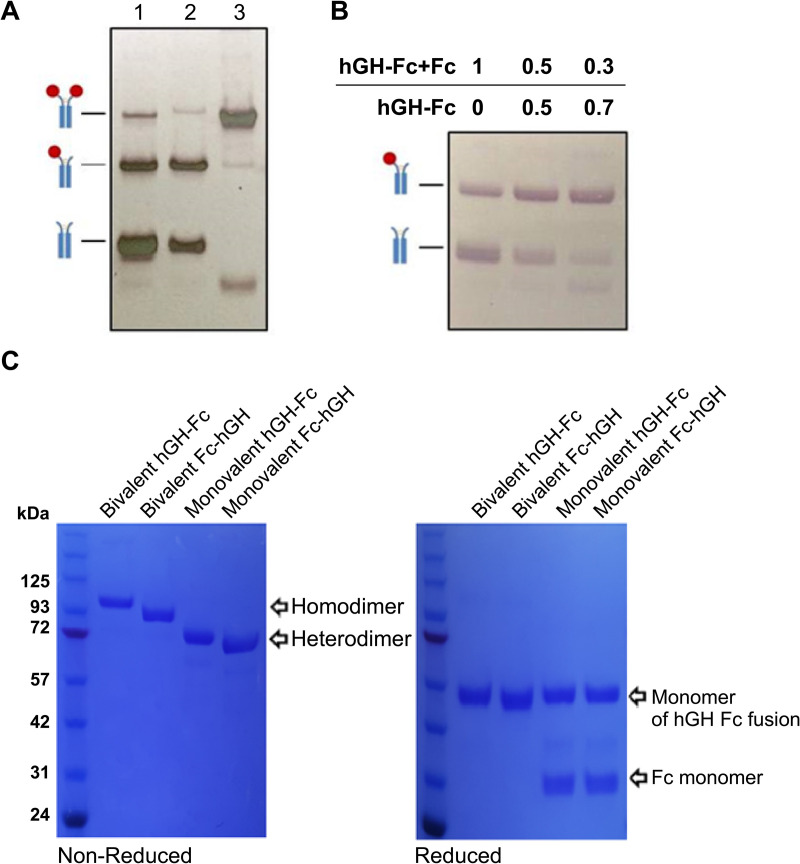
Expressed and purified hGH-Fc fusion proteins. **(A)** Anti-Fc western blot analysis of hGH-Fc fusion proteins and byproducts in transient Chinese hamster ovary (CHO) cell expression supernatants. Lane 1: monovalent hGH-Fc with wild-type CH3 Fc domains. Lane 2: monovalent hGH-Fc with knob and hole mutations in the CH3 Fc domains. Lane 3: bivalent hGH-Fc. The expected band identities are indicated on the side of the blot. **(B)** Anti-Fc western blot analysis of hGH-Fc fusion proteins expressed by co-transfection with two plasmids at various ratios. The ratios of the co-transfected plasmids are indicated above the blot. **(C)** Sodium dodecyl-polyacrylamide gel electrophoresis (SDS-PAGE) of bivalent fusion proteins purified using protein A chromatography and monovalent hGH-Fc fusion proteins purified using hGH affinity chromatography followed by hydrophobic interaction chromatography. Original blot and gel images are provided in S1 raw image.

Analysis of culture supernatants showed that bivalent hGH fusion constructs were expressed mainly as the expected homodimeric form (Fig 2A, lane 3). In contrast, monovalent constructs were expressed as a heterogeneous mixture of chain combinations, including hGH-Fc dimers, Fc dimers, and the desired monovalent hGH-Fc form (Fig 2A, lane 1).

Knob (T366W) and hole (T366S, L368A, Y407V) mutations in the CH3 Fc domains were introduced to promote heterodimerization, which reduced homodimeric product levels (Fig 2A, lane 2). However, the Fc dimer remained a major byproduct. Co-transfection with an additional plasmid expressing only the hGH-Fc chain alongside the plasmid expressing both hGH-Fc and Fc chains further reduced Fc dimer levels (Fig 2B), suggesting that excess Fc chain expression contributed to the formation of Fc dimers.

SDS-PAGE analysis revealed that purified bivalent constructs existed as dimers under non-reducing conditions and as monomers under reducing conditions. Purified monovalent constructs existed as monomeric Fc linked to monomeric hGH under reducing conditions. Bivalent constructs achieved satisfactory purity with protein A chromatography alone, while monovalent constructs required additional chromatographic steps for high purity (Fig 2C).

### *In vitro* activity of various hGH-Fc fusion constructs

**Fig 3:**
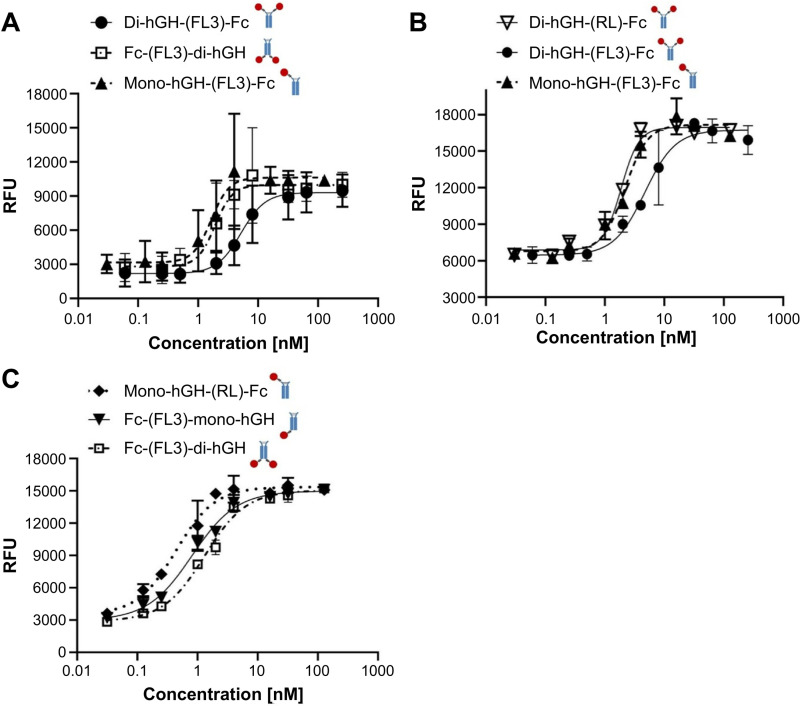
*In vitro* Nb2 cell-based activity assay results for various hGH-Fc fusion proteins. Schematic diagrams to the right of each sample name represent the protein structure of the corresponding construct, with hGH moieties (circle) and Fc regions (rectangular) connected by linkers (wavy line). **(A)** Comparison of fusion position and valency effects: Di-hGH-(FL3)-Fc (bivalent, N-terminal fusion), Fc-(FL3)-di-hGH (bivalent, C-terminal fusion), and Mono-hGH-(FL3)-Fc (monovalent, N-terminal fusion). **(B)** Comparison of linker rigidity and valency effects: Di-hGH-(RL)-Fc (bivalent with rigid linker), Di-hGH-(FL3)-Fc (bivalent with flexible linker), and Mono-hGH-(FL3)-Fc (monovalent with flexible linker). **(C)** Comparison of optimized constructs: Fc-(FL3)-di-hGH (bivalent, C-terminal fusion), Fc-(FL3)-mono-hGH (monovalent, C-terminal fusion), and Mono-hGH-(RL)-Fc (monovalent, N-terminal fusion with rigid linker). Data are presented as the mean ± standard deviation (SD).

The *in vitro* activity of hGH-Fc fusion proteins was influenced by the position and valency of the hGH moiety. Bivalent Fc-hGH constructs (EC_50_ = 1.91 nM) showed 2.7-fold higher activity than bivalent hGH-Fc constructs (EC_50_ = 5.19 nM). Monovalent hGH-Fc fusion proteins (EC_50_ = 1.64 nM) had activity similar to Fc-hGH when analyzed on a whole-molecule basis. However, monovalent constructs showed 1.7-fold higher activity per hGH moiety than Fc-hGH and 6.3-fold higher activity than hGH-Fc, likely due to their single hGH moiety per molecule (Fig 3A).

The characteristics of the linker connecting the hGH moiety and the Fc region also affected *in vitro* activity. Bivalent hGH-Fc constructs with a rigid alpha-helical A(EAAAK)_5_A linker demonstrated 2.5-fold higher activity (EC_50_ = 1.87 nM) compared with bivalent constructs with a flexible (GGGGS)_3_ linker (EC_50_ = 4.68 nM) (Fig 3B). Combining these favorable factors resulted in increased activity. Monovalent hGH-Fc with a rigid linker (EC_50_ = 0.44 nM) and monovalent Fc-hGH (EC_50_ = 0.81 nM) showed significantly higher activity than bivalent Fc-hGH (EC_50_ = 1.31 nM) (Fig 3C).

### Pharmacokinetics of various hGH-Fc fusion constructs in rats

The PK profiles of the rhGH and hGH-Fc fusion proteins in SD rats are summarized in Table 2. While rhGH was rapidly cleared after subcutaneous administration, Fc fusion proteins exhibited prolonged serum concentrations. However, the C_max_, AUC, and half-lives varied significantly depending on the construct design.

**Table 2:** Mean (± standard deviation) pharmacokinetic parameters after a single subcutaneous administration of recombinant human growth hormone (rhGH) or hGH-Fc fusion proteins in rats.

Test article	Dose	C_max_ (ng/mL)	T_max_ (day)	AUC_0-∞_ (day ng/mL)	t_1/2_ (day)
Experiment A					
rhGH	0.2 mg/kg	38.5 ± 2.7	0.033 ± 0.011		0.026 ± 0.008
Di-hGH-(FL3)-Fc	1 mg/kg	222.8 ± 30.4††	0.567 ± 0.401*	582.9. ± 63.4***	1.50 ± 0.07
Fc-(FL3)-di-hGH	1 mg/kg	434.3 ± 124.6**	0.167 ± 0.000	219.9 ± 61.5	3.06 ± 0.31***
Experiment B					
Di-hGH-(FL3)-Fc	1 mg/kg	178.7 ± 19.8	0.533 ± 0.431	474.3. ± 33.9†††	1.89 ± 0.64
Mono-hGH-(FL3)-Fc	1 mg/kg	795.7 ± 110.7***	0.700 ± 0.415	1019.0 ± 102.7***	1.43 ± 0.21
Di-hGH-(RL)-Fc	1 mg/kg	150.8 ± 48.1	0.167 ± 0.000	133.5 ± 25.0	1.79 ± 0.46
Experiment C					
Di-hGH-(FL3)-Fc	1 mg/kg	222.2 ± 122.7	0.400 ± 0.346	452.5 ± 106.8	1.80 ± 0.11†††
Fc-(FL3)-mono-hGH	1 mg/kg	1654.9 ± 70.0***	1.000 ± 0.000*	1781.0 ± 324.7***	1.70 ± 0.38††
Mono-hGH-(RL)-Fc	1 mg/kg	318.8 ± 85.1	0.375 ± 0.370	361.3 ± 81.6	0.91 ± 0.20
Experiment D					
Di-hGH-(FL3)-Fc	1 mg/kg	364.7 ± 38.5†	0.200 ± 0.075	544.5 ± 95.0†	1.14 ± 0.27
Di-hGH-(GL)-Fc	1 mg/kg	24.6 ± 16.1	0.150 ± 0.037	16.9 ± 7.5	0.93 ± 0.22
Mono-hGH-(FL3)-Fc	1 mg/kg	1285.0 ± 251.2***	0.867 ± 0.298***	1315.7 ± 508.8***	1.57 ± 0.27*
Mono-hGH-(GL)-Fc	1 mg/kg	85.2 ± 37.9	0.150 ± 0.037	55.7 ± 11.1	0.75 ± 0.14

* p < 0.05, ** p < 0.01, *** p < 0.001 (vs. all other constructs in each experiment group).

† p < 0.05 (vs. Di-hGH-(GL)-Fc and Mono-hGH-(GL)-Fc for Cmax and AUC).

†† p < 0.01 (vs. rGH for Cmax in experiment A and vs. Mono-hGH-(RL)-Fc for t_1/2_ in experiment C).

††† p < 0.001 (vs. Di-hGH-(RL)-Fc for AUC in experiment B and vs. mono-hGH-(RL)-Fc for t_1/2_ in experiment C).

**Fig 4:**
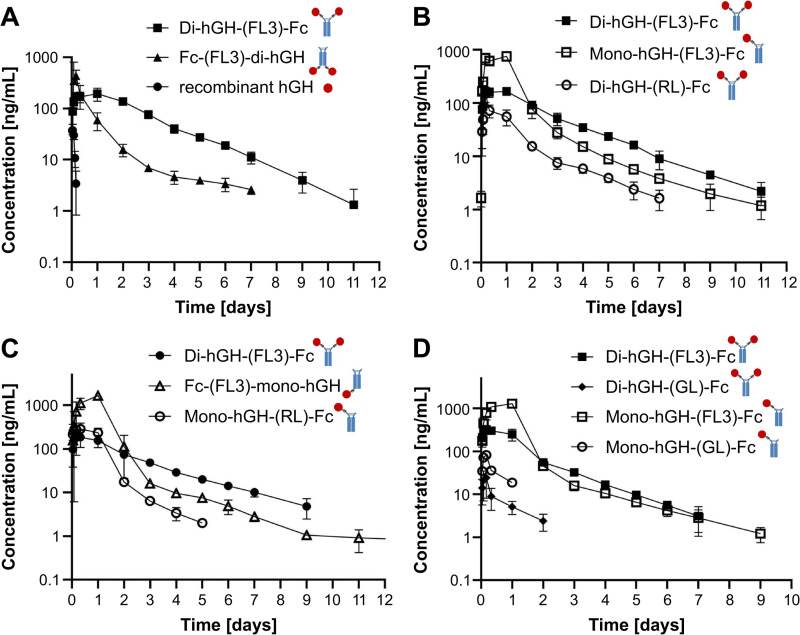
Pharmacokinetics of recombinant human growth hormone and hGH-Fc fusion proteins in Sprague–Dawley rats. Recombinant human growth hormone (rhGH; 0.2 mg/kg) or hGH-Fc fusion proteins (1 mg/kg) were administered by single-dose subcutaneous injections. Serum concentrations were determined using a commercial hGH ELISA kit with custom standard curves for each fusion protein. Pharmacokinetic parameters were calculated using non-compartmental analysis in WinNonlin software. The pharmacokinetic parameters of **(A)**, **(B)**, **(C)**, and **(D)** correspond to Experiments A, B, C, and D in Table 2, respectively. Data are presented as the mean ± standard deviation (SD).

Bivalent Fc-hGH with a flexible (GGGGS)_3_ linker had a higher C_max_ but showed rapid clearance and a lower AUC compared to bivalent hGH-Fc with the same linker (Fig 4A). Constructs with rigid A(EAAAK)_5_A linkers had lower AUCs than those with flexible (GGGGS)_3_ linkers. Monovalent constructs (hGH-Fc and Fc-hGH) with flexible linkers showed higher initial serum levels, C_max_, and AUC than bivalent hGH-Fc but declined rapidly during the distribution phase, leading to lower serum levels after day 2 (Fig 4B, 4C). Glycosylated flexible linker constructs exhibited lower C_max_ and AUC values with faster clearance compared with their non-glycosylated counterparts (Fig 4D).

### Pharmacodynamic evaluation in hypophysectomized rats

**Fig 5:**
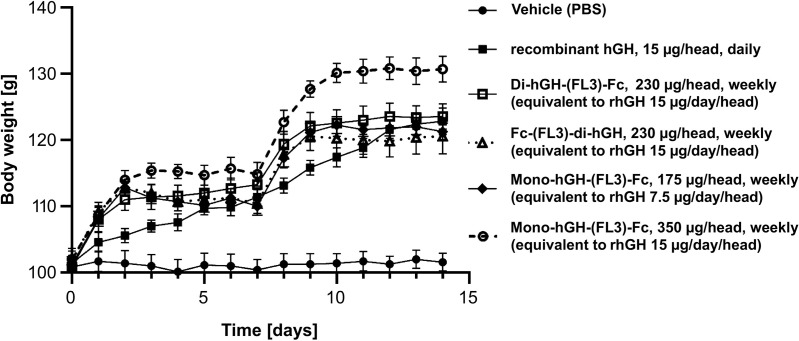
Body weight gain of hypophysectomized rats treated with vehicle, rhGH, or hGH-Fc fusion proteins. Rats received daily dosing of the vehicle or rhGH (days 0–13) or weekly dosing (days 0 and 7) of hGH-Fc fusion proteins for 2 weeks. Data are presented as the mean ± standard error of the mean (SEM). All hGH or hGH-Fc fusion protein-treated groups showed significant (p < 0.001) increases in body weight compared with the vehicle (PBS) group (⏺). Monovalent hGH-Fc administered at 350 µg/head/week (○) showed significantly (p < 0.05) higher body weight gain than the rhGH group (⏹).

Animals receiving daily rhGH demonstrated steady weight gain throughout the dosing period, whereas vehicle-treated animals did not exhibit any weight gain. All groups treated with hGH-Fc fusion proteins showed significant weight gain compared to the vehicle group (Fig 5). In the hGH-Fc fusion protein-treated groups, body weight increased rapidly during the first 2 days after injection, followed by a plateau until the next injection. This pattern reflects the PK profile observed in rats, where the AUC during the first 2 days after injection accounted for approximately 80% of the total AUC.

A comparison of monovalent and bivalent hGH-Fc fusion proteins at equimolar doses revealed that monovalent constructs exhibited higher body weight gain, suggesting greater activity per hGH moiety in the monovalent form. Monovalent hGH-Fc contains only one hGH moiety per molecule, resulting in half the hGH content of bivalent hGH-Fc. However, when the doses were normalized for hGH content, monovalent hGH-Fc (350 µg/head/week) still induced significantly greater weight gain than bivalent hGH-Fc (230 µg/head/week).

**Fig 6:**
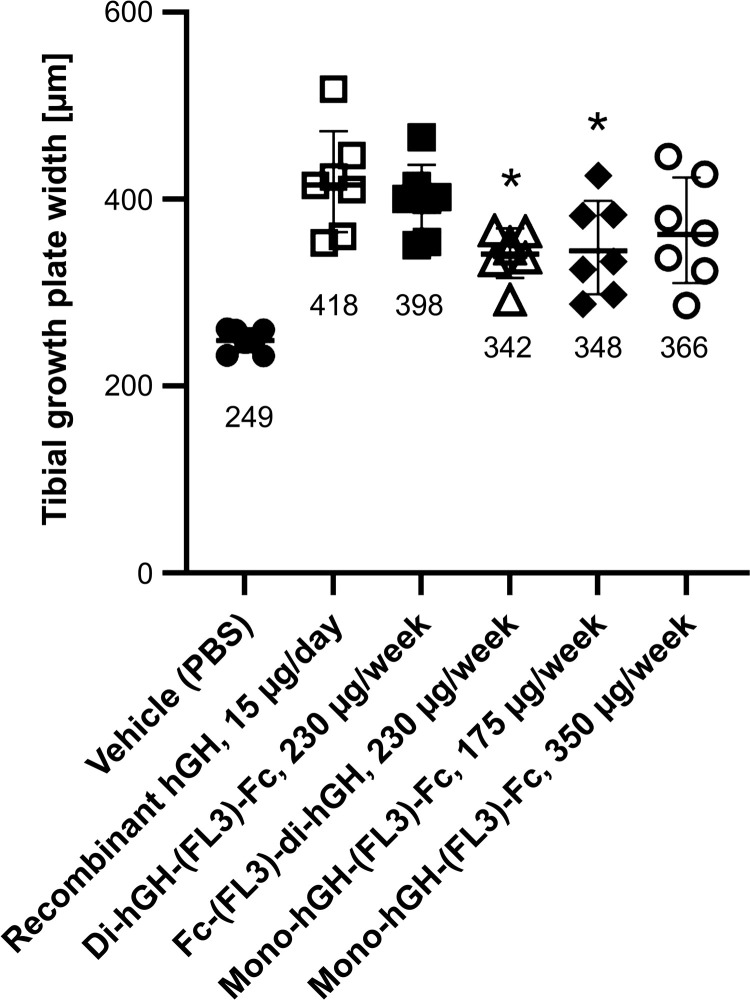
Tibial growth plate widths of hypophysectomized rats. After evaluating body weight gain, the hypophysectomized rats were euthanized, and tibial growth plate widths were measured. The numbers in the graph indicate the mean values for each group. All hGH or hGH-Fc fusion protein-treated groups showed significant (p < 0.01) increases in growth plate width compared to the vehicle (PBS) group. Asterisks (*) indicate significantly lower mean values (p < 0.05) compared with those of the rhGH group. Di-hGH-(FL3)-Fc (230 ug/week) and mono-hGH-(FL3)-Fc (350 ug/week) treatment group showed no significant differences with the rhGH group. No significant differences were observed among the hGH-Fc fusion proteins.

The administration of rhGH or hGH-Fc fusion proteins also significantly increased tibial growth plate width compared with vehicle controls (Fig 6). Monovalent hGH-Fc administered at 350 µg/head/week resulted in the highest body weight gain but did not show significantly greater growth plate width compared to other hGH fusion proteins or rhGH-treated groups. This indicates that growth plate width did not correlate directly with body weight gain in these treatment groups.

### Evaluation of hGH-Fc with varying linker lengths

In the rat PK experiments, bivalent hGH-Fc with a flexible linker demonstrated a favorable profile in terms of *in vivo* longevity, characterized by a non-exaggerated C_max_ and consistent clearance throughout the observation period. This contrasted with other constructs that either had low overall AUC or high AUC but underwent rapid elimination during the distribution phase. To further evaluate the effects of linker length on *in vitro* and *in vivo* characteristics, additional bivalent hGH-Fc constructs were analyzed using an Nb2 cell-based assay, rat PK studies, and hypophysectomized rat experiments.

**Fig 7:**
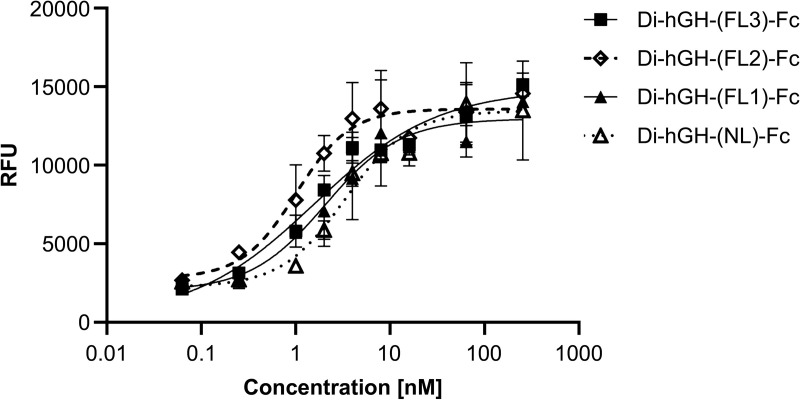
*In vitro* Nb2 cell-based activity of bivalent hGH-Fc fusion proteins with various linker lengths. Constructs tested include Di-hGH-(NL)-Fc (no linker), Di-hGH-(FL1)-Fc (one GGGGS unit), Di-hGH-(FL2)-Fc (two GGGGS units), and Di-hGH-(FL3)-Fc (three GGGGS units). Data are presented as mean ± standard deviation (SD).

In the *in vitro* activity comparison, bivalent hGH-Fc fusion proteins with linker lengths ranging from no linker (i.e., direct fusion of hGH and Fc) to triplicate GGGGS units were tested. Constructs without a linker (di-hGH-(NL)-Fc) exhibited lower activity compared to those with two or three GGGGS units (Fig 7). However, no consistent trend in activity was observed with increasing linker length.

**Fig 8:**
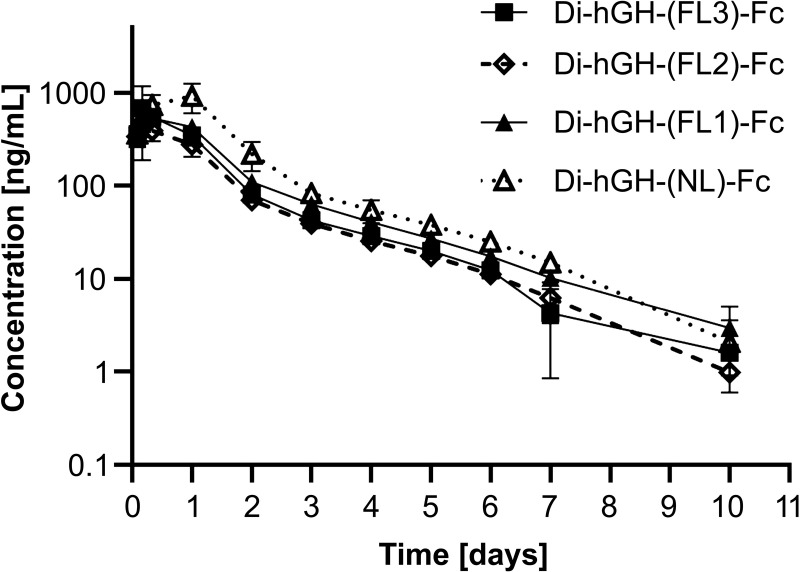
Pharmacokinetics of bivalent hGH-Fc fusion proteins with various linker lengths in Sprague–Dawley rats. Bivalent hGH-Fc fusion proteins (1 mg/kg) were administered by single-dose subcutaneous injections. Data are presented as the mean ± standard deviation (SD).

The PK profiles of bivalent hGH-Fc constructs with varying linker lengths are shown in Fig 8 and Table 3. Constructs with shorter linker lengths, such as di-hGH-(NL)-Fc and di-hGH-(FL1)-Fc (containing zero or one GGGGS unit, respectively), exhibited higher AUCs than did those with two or three GGGGS units. Among them, the construct without a linker (di-hGH-(NL)-Fc) showed a significantly higher AUC (p < 0.001) than the other constructs.

**Table 3:** Mean (± standard deviation) pharmacokinetic parameters after a single subcutaneous administration of bivalent hGH-Fc fusion proteins with various linker lengths in rats.

Test article	Dose	C_max_ (ng/mL)	T_max_ (day)	AUC_0-∞_ (day ng/mL)	Terminal t_1/2_ (day)
Di-hGH-(FL3)-Fc	1 mg/kg	755.6 ± 461.4	0.300 ± 0.075†	788.4 ± 112.6	1.30 ± 0.38
Di-hGH-(FL2)-Fc	1 mg/kg	420.4 ± 74.9	0.200 ± 0.075	627.2 ± 127.8	1.52 ± 0.30
Di-hGH-(FL1)-Fc	1 mg/kg	533.8 ± 44.1	0.333 ± 0.000††	909.6 ± 104.7	1.55 ± 0.11
Di-hGH-(NL)-Fc	1 mg/kg	1012.4 ± 184.8†	1.000 ± 0.000***	1634.7 ± 261.7***	1.650.32

*** p < 0.001 (vs. all other constructs).

† p < 0.05 (vs. Di-hGH-(FL1)-Fc and Di-hGH-(FL2)-Fc for Cmax, and vs. Di-hGH-(FL2)-Fc for Tmax).

†† p < 0.01 (vs.Di-hGH-(FL2)-Fc for Tmax).

**Fig 9:**
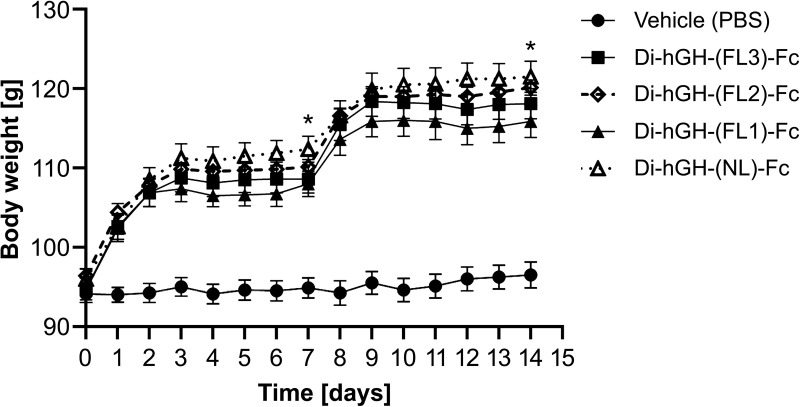
Body weight gain of hypophysectomized rats treated with hGH-Fc fusion proteins with various linker lengths. Rats received weekly dosing (days 0 and 7) of hGH-Fc fusion proteins with various linker lengths or vehicle control (PBS). Data are presented as the mean ± standard error of the mean (SEM). Asterisks (*) indicate a significant (p < 0.05) increase in body weight of the Di-hGH-(NL)-Fc compared to other hGH-Fc constructs. All hGH-Fc fusion protein-treated groups showed a significant (P < 0.001) increase in body weight compared to the vehicle (PBS) group.

In the hypophysectomized rat efficacy evaluation, constructs with shorter linkers also exhibited superior performance. The no-linker construct (di-hGH-(NL)-Fc), which had the highest AUC in the rat PK study, produced the greatest body weight gain. By day 7, rats treated with di-hGH-(NL)-Fc exhibited significantly higher body weight gain (p < 0.05) than those treated with all other hGH-Fc fusion proteins. This trend continued through day 14, with the di-hGH-(NL)-Fc group maintaining significantly higher weight gain (p < 0.05), compared with the group treated with di-hGH-(FL1)-Fc, which contained one linker unit (Fig 9).

## Discussion

The remarkable stability and dimeric structure of the IgG Fc region provide versatility in designing Fc fusion proteins to enhance *in vivo* half-life. This versatility enables modifications that can adjust the activity and PK/PD properties of the fusion protein. In this study, we explored key structural features, including linker type, effector moiety valency, and fusion position, to evaluate their effects on the PK and PD properties of hGH-Fc fusion proteins.

For this study, transient expression systems were used to produce Fc fusion proteins rather than stable cell lines optimized for individual Fc chain expression. Although transient expression limited insights into manufacturability, heterodimeric (monovalent) constructs were more challenging to produce due to the co-expression of homodimeric impurities, such as Fc dimers. These impurities co-purified during protein A chromatography, necessitating additional purification steps and reducing overall yield. Such challenges may pose economic obstacles for the commercial production of heterodimeric Fc fusion proteins, particularly for cost-sensitive treatments like hGH replacement therapy.

Structural modifications designed to increase spatial separation between domains and reduce steric hindrance enhanced *in vitro* activity. For example, constructs with rigid, alpha-helix-forming linkers (A(EAAAK)_5_A) demonstrated higher *in vitro* activity than did constructs using flexible (GGGGS)_3_ linkers. These findings align with those of Zhao et al., who compared alpha-helical and flexible linkers in HSA-IFN-alpha2b fusion proteins [24]. Monovalent hGH-Fc fusion proteins also exhibited higher *in vitro* activity per molecule, compared with bivalent constructs, despite containing only half the number of effector moieties. This suggests that the proximity of the two hGH moieties in bivalent constructs may interfere with receptor binding.

The fusion position significantly influenced *in vitro* activity. Fusion proteins with hGH located at the N-terminal (Fc-hGH) exhibited approximately threefold higher *in vitro* activity compared with those with hGH at the C-terminal (hGH-Fc). Similar observations have been reported in XTEN-hGH [25] and albumin-hGH [26] fusion proteins, where N-terminal fusion was less detrimental to hGH activity than C-terminal fusion.

The PK profiles of hGH-Fc fusion proteins varied significantly depending on linker type, valency, and fusion position. Despite all constructs exceeding the molecular weight threshold for renal filtration and sharing the IgG Fc region for FcRn-mediated half-life extension, their PK profiles differed substantially. Constructs such as bivalent Fc-hGH with a flexible (GGGGS)_3_ linker and bivalent hGH-Fc with a rigid A(EAAAK)_5_A linker exhibited higher *in vitro* activity but lower *in vivo* exposure compared to bivalent hGH-Fc with a flexible (GGGGS)_3_ linker. In contrast, bivalent hGH-Fc without a linker exhibited lower *in vitro* activity but higher *in vivo* exposure than constructs with flexible linkers. Monovalent constructs with flexible (GGGGS)_3_ linkers exhibited higher C_max_ and AUC values but experienced rapid clearance during the distribution phase, resulting in lower serum levels after day 2. These findings suggest a potential relationship between *in vitro* activity and hGH receptor-mediated clearance, consistent with observations by Cleland et al. [25].

The widely differing PK profiles observed across the constructs in this study do not indicate a straightforward relationship between *in vitro* activity and PK outcomes. For example, monovalent hGH-Fc with a flexible linker, bivalent hGH-Fc with a rigid linker, and bivalent Fc-hGH with a flexible linker all displayed similar EC_50_ values in Nb2 cell-based assays. However, monovalent hGH-Fc constructs showed significantly higher AUC values compared with their bivalent counterparts, suggesting that additional factors—such as differences in the efficiency of subcutaneous transport or FcRn-mediated endosomal recycling—may influence construct bioavailability. Despite all constructs having similar charges (pI values of approximately 5.5), monovalent constructs demonstrated higher C_max_ and AUC values than bivalent constructs with comparable *in vitro* activity. These findings imply that other physicochemical properties, such as molecular weight or structural shape, may play a critical role in interstitial transport from the subcutaneous administration site and subsequent lymphatic uptake into the systemic circulation.

Incorporating N-glycosylation sites into the linker can increase rigidity and stability [27]. In this study, constructs with glycosylated linkers exhibited faster clearance than non-glycosylated counterparts (Fig 4D). Based on our glycan analysis, these constructs predominantly contained non-sialylated N-glycans (S2 Fig, S2–S5 Tables). While our study was not designed to mechanistically link glycan composition to clearance, prior work has demonstrated that non-sialylated glycans interact with hepatic asialoglycoprotein receptors, accelerating clearance [28–31]. This mechanism has been well-documented for other glycoproteins, though further studies with targeted experiments such as sialylated variants of these constructs or receptor-blocking assays are required to confirm this hypothesis for our specific hGH-Fc constructs.

The superior *in vitro* activity of monovalent hGH-Fc constructs was corroborated in hypophysectomized rat experiments, where monovalent constructs resulted in approximately 30% higher net body weight gain at equivalent hGH-normalized doses compared with bivalent constructs. For bivalent constructs, differences in *in vivo* potency based on linker length were less pronounced, possibly due to offsetting effects of faster clearance and higher molecular activity. However, constructs such as hGH-Fc directly fused to the Fc hinge region, which exhibited high AUC but low *in vitro* activity, also showed greater body weight gain. While monovalent constructs with high C_max_ and AUC values demonstrated promising activity, these properties may increase the risks of lipoatrophy [32] or supraphysiological IGF-1 responses, which are undesirable for long-acting hGH therapies.

Based on our findings, monovalent hGH-Fc fusion proteins with flexible linkers demonstrated superior *in vivo* potency, inducing greater body weight gain at equivalent hGH-normalized doses compared to bivalent constructs. However, their rapid clearance during the distribution phase suggests a need for further optimization to enhance longevity. Among bivalent constructs, hGH-Fc without a linker exhibited the highest AUC values and significant body weight gain but showed reduced *in vitro* activity due to potential steric hindrance affecting receptor binding. Bivalent hGH-Fc with flexible linkers provided a balanced PK/PD profile with sustained serum levels and moderate AUC values, avoiding risks associated with excessive Cmax values. Considering these factors, monovalent hGH-Fc fusion proteins and bivalent hGH-Fc without linkers emerge as promising candidates for continued development.

A limitation of this study is the primary focus on body weight gain and tibial growth plate width as PD endpoints. Additional markers of GH activity, such as IGF-1 levels or metabolic parameters, were not assessed. Extended observations, including regular IGF-1 monitoring and local toxicology studies, are necessary to evaluate potential risks, such as lipoatrophy or supraphysiological. Our study demonstrated prolonged serum concentrations of hGH-Fc fusion proteins in Sprague–Dawley rats. However, we recognize that this model has limitations in predicting human PK due to differences in FcRn binding specificities between rodents and humans [33,34]. Transgenic models expressing human FcRn, such as Tg32 mice, have been validated for predicting clinical PK profiles of therapeutic antibodies and Fc fusion proteins by minimizing species-specific biases [35,36] This model enabled comparative analysis of structural variants and provided initial proof-of-concept for design dependent PK variability, human FcRn transgenic mice models would better resolve subtle differences in FcRn-mediated recycling and predict clinical PK due to species-specific FcRn interaction. Future studies should utilize transgenic mice expressing human FcRn to more accurately assess the longevity and translational potential of these constructs.

## Conclusion

In summary, this study highlights the significant impact of structural design choices on the PK and PD properties of hGH-Fc fusion proteins. Our findings provide a foundation for optimizing Fc fusion protein designs to enhance the potency and longevity of long-acting therapeutic proteins. However, the poor predictability of rodent subcutaneous PK data for human outcomes [37] underscores the need for improved animal models or early clinical studies to support future developments.

## Supporting information

S1 Fig Liquid chromatography-mass spectrometric analysis of site-specific N-glycosylation in reduced monomeric Di-hGH-(GS3)-Fc fusion proteins with engineered glycosylation sites at different positions in the GS3 linker. Electrospray ionization-time of flight mass spectrometry coupled with reversed-phase high-performance liquid chromatography was used to analyze intact proteins. All constructs were reduced with 50 mM dithiothreitol and analyzed in their monomeric state. Detailed GS3 linker sequences for each protein construct are provided in S1 Table. **(A)** Control Di-hGH-(GS3)-Fc without engineered glycosylation sites showed baseline glycosylation with N-glycans only at the conserved Fc glycosylation site. **(B)** Di-hGH-(GS3)-Fc_Glyco1, with an N-glycosylation site proximal to hGH, displayed minimal glycosylation. **(C)** Di-hGH-(GS3)-Fc_Glyco2, with a middle-positioned N-glycosylation site, showed moderate glycosylation. **(D)** Di-hGH-(GS3)-Fc_Glyco3, with an N-glycosylation site distal to hGH, exhibited the highest glycosylation efficiency. Major glycoforms were assigned by matching experimentally determined masses to theoretical masses calculated using data from S1 and S2 Tables. Schematic representations of glycoprotein structures with one or two N-glycans are shown for each construct. The x-axis represents deconvoluted mass (amu), while the y-axis shows relative intensity (counts).
(DOCX)

S2 Fig Liquid chromatography-mass spectrometry analysis of hGH-Fc fusion protein constructs with a linker containing three engineered N-glycosylation sites. Human growth hormone (hGH) Fc fusion protein constructs with engineered glycosylation sites in the linker region. Two constructs, Mono-hGH-(GL)-Fc **(A)** and Di-hGH-(GL)-Fc **(B)**, produced in Chinese hamster ovary (CHO) cells, were subjected to glycan profiling at the intact protein level. To reduce spectral complexity from oligosaccharide pairs, Di-hGH-(GL) was treated with 50 mM dithiothreitol. Major peaks in the mass spectra were assigned to specific glycan species attached to the protein constructs. Both constructs exhibited heterogeneity in glycoform composition and glycan site occupancy. Notably, no sialylation was observed in the analyzed samples. A schematic representation of the most probable glycosylated construct structures is shown above each mass spectrum for visual reference.
(DOCX)

S1 Table Theoretical masses and linker sequences of reduced monomeric Di-hGH-(GS3)-Fc fusion protein constructs containing engineered N-glycosylation sites. This table provides the construct names, linker sequences, and calculated theoretical masses of reduced monomeric Di-hGH-(GS3)-Fc fusion proteins after dithiothreitol treatment. The control construct contains three GGGGS sequence repeats (GS3 linker), while glycoengineered constructs (Glyc1, Glyc2, and Glyc3) feature an N-glycosylation sequon (NGT) at different positions within the GS3 linker: the first repeat (Glyc1), second repeat (Glyc2), and third repeat (Glyc3). Differences in theoretical mass between the control and glycoengineered constructs reflect the amino acid substitutions required to introduce the N-glycosylation sites.
(DOCX)

S2 Table Glycan species of hGH-Fc fusion protein constructs with glycosylated linkers. This table presents the theoretical mass data for various glycan species found in hGH-Fc fusion protein constructs with glycosylated linkers. The glycan structures are represented using symbols: ◇ Sialic acid, ○ Galactose, □ GlcNAc, ● Mannose, △ Fucose. The table includes the glycan composition and the corresponding theoretical mass for each glycan species.
(DOCX)

S3 Table Glycan species of hGH-Fc fusion protein constructs with glycosylated linkers. This table presents the theoretical masses of non-glycosylated hGH-Fc fusion protein backbones for both monomeric Di-hGH-(GL)-Fc and heterodimeric Mono-hGH-(GL)-Fc forms.
(DOCX)

S4 Table Glycan species of hGH-Fc fusion protein constructs with glycosylated linkers. This table lists the theoretical masses of individual monosaccharides that compose the glycan structures found in the fusion proteins.
(DOCX)

S5 Table Glycan species of hGH-Fc fusion protein constructs with glycosylated linkers. This table summarizes the glycan species of hGH-Fc fusion protein constructs with glycosylated linkers. Theoretical masses of glycosylated hGH-Fc fusion proteins were calculated using data from S2–S4 Tables to assign major peaks observed in S2 Fig to specific glycan species. These predictions were based on matching experimentally determined peak masses from S2 Fig to the theoretical masses of glycosylated constructs.
(DOCX)

S1 raw image Original western blot and SDS-PAGE gel images for protein characterization. (PDF)

S1 raw data Dataset for *In Vitro* activity (EC50/RFU), pharmacokinetic parameters, and hypophysectomized rat efficacy studies of hGH-Fc constructs. (XLSX)
