# Supplementary material for: Engineering of long-acting human growth hormone-Fc fusion proteins: Effects of valency, fusion position, and linker design on pharmacokinetics and efficacy
Source: PLoS One. 2025 May 15;20(5):e0323791. doi: 10.1371/journal.pone.0323791 (PMC12080763; doi:10.1371/journal.pone.0323791)
Supplement: S1 Fig — Electrospray ionization-time of flight mass spectrometry coupled with reversed-phase high-performance liquid chromatography was used to analyze intact proteins. All constructs were reduced with 50 mM dithiothreitol and analyzed in their monomeric state. Detailed GS3 linker sequences for each protein construct are provided in S1 Table. (A) Control Di-hGH-(GS3)-Fc without engineered glycosylation sites showed baseline glycosylation with N-glycans only at the conserved Fc glycosylation site. (B) Di-hGH-(GS3)-Fc_Glyco1, with an N-glycosylation site proximal to hGH, displayed minimal glycosylation. (C) Di-hGH-(GS3)-Fc_Glyco2, with a middle-positioned N-glycosylation site, showed moderate glycosylation. (D) Di-hGH-(GS3)-Fc_Glyco3, with an N-glycosylation site distal to hGH, exhibited the highest glycosylation efficiency. Major glycoforms were assigned by matching experimentally determined masses to theoretical masses calculated using data from S1 and S2 Tables. Schematic representations of glycoprotein structures with one or two N-glycans are shown for each construct. The x-axis represents deconvoluted mass (amu), while the y-axis shows relative intensity (counts). (DOCX) [file pone.0323791.s001.docx]

**
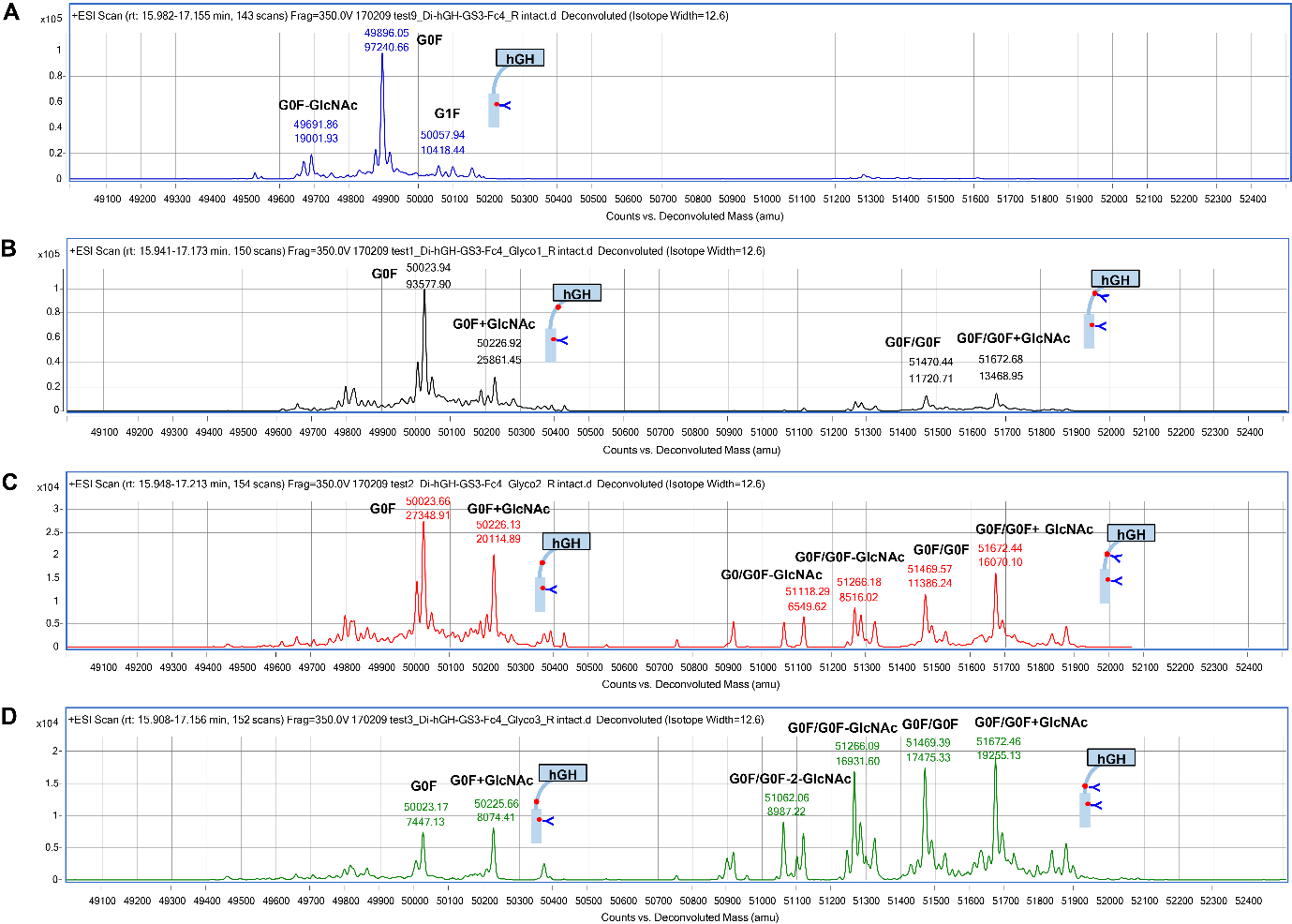
**

**S1 Fig. Liquid chromatography-mass spectrometric analysis of site-specific N-glycosylation in reduced monomeric Di-hGH-(GS3)-Fc fusion proteins with engineered glycosylation sites at different positions in the GS3 linker.** Electrospray ionization-time of flight mass spectrometry coupled with reversed-phase high-performance liquid chromatography was used to analyze intact proteins. All constructs were reduced with 50 mM dithiothreitol and analyzed in their monomeric state. Detailed GS3 linker sequences for each protein construct are provided in S1 Table. (A) Control Di-hGH-(GS3)-Fc without engineered glycosylation sites showed baseline glycosylation with N-glycans only at the conserved Fc glycosylation site. (B) Di-hGH-(GS3)-Fc_Glyco1, with an N-glycosylation site proximal to hGH, displayed minimal glycosylation. (C) Di-hGH-(GS3)-Fc_Glyco2, with a middle-positioned N-glycosylation site, showed moderate glycosylation. (D) Di-hGH-(GS3)-Fc_Glyco3, with an N-glycosylation site distal to hGH, exhibited the highest glycosylation efficiency. Major glycoforms were assigned by matching experimentally determined masses to theoretical masses calculated using data from S1 and S2 Tables. Schematic representations of glycoprotein structures with one or two N-glycans are shown for each construct. The x-axis represents deconvoluted mass (amu), while the y-axis shows relative intensity (counts).
