# Supplementary material for: Engineering of long-acting human growth hormone-Fc fusion proteins: Effects of valency, fusion position, and linker design on pharmacokinetics and efficacy
Source: PLoS One. 2025 May 15;20(5):e0323791. doi: 10.1371/journal.pone.0323791 (PMC12080763; doi:10.1371/journal.pone.0323791)
Supplement: S2 Fig — Human growth hormone (hGH) Fc fusion protein constructs with engineered glycosylation sites in the linker region. Two constructs, Mono-hGH-(GL)-Fc (A) and Di-hGH-(GL)-Fc (B), produced in Chinese hamster ovary (CHO) cells, were subjected to glycan profiling at the intact protein level. To reduce spectral complexity from oligosaccharide pairs, Di-hGH-(GL) was treated with 50 mM dithiothreitol. Major peaks in the mass spectra were assigned to specific glycan species attached to the protein constructs. Both constructs exhibited heterogeneity in glycoform composition and glycan site occupancy. Notably, no sialylation was observed in the analyzed samples. A schematic representation of the most probable glycosylated construct structures is shown above each mass spectrum for visual reference. (DOCX) [file pone.0323791.s002.docx]

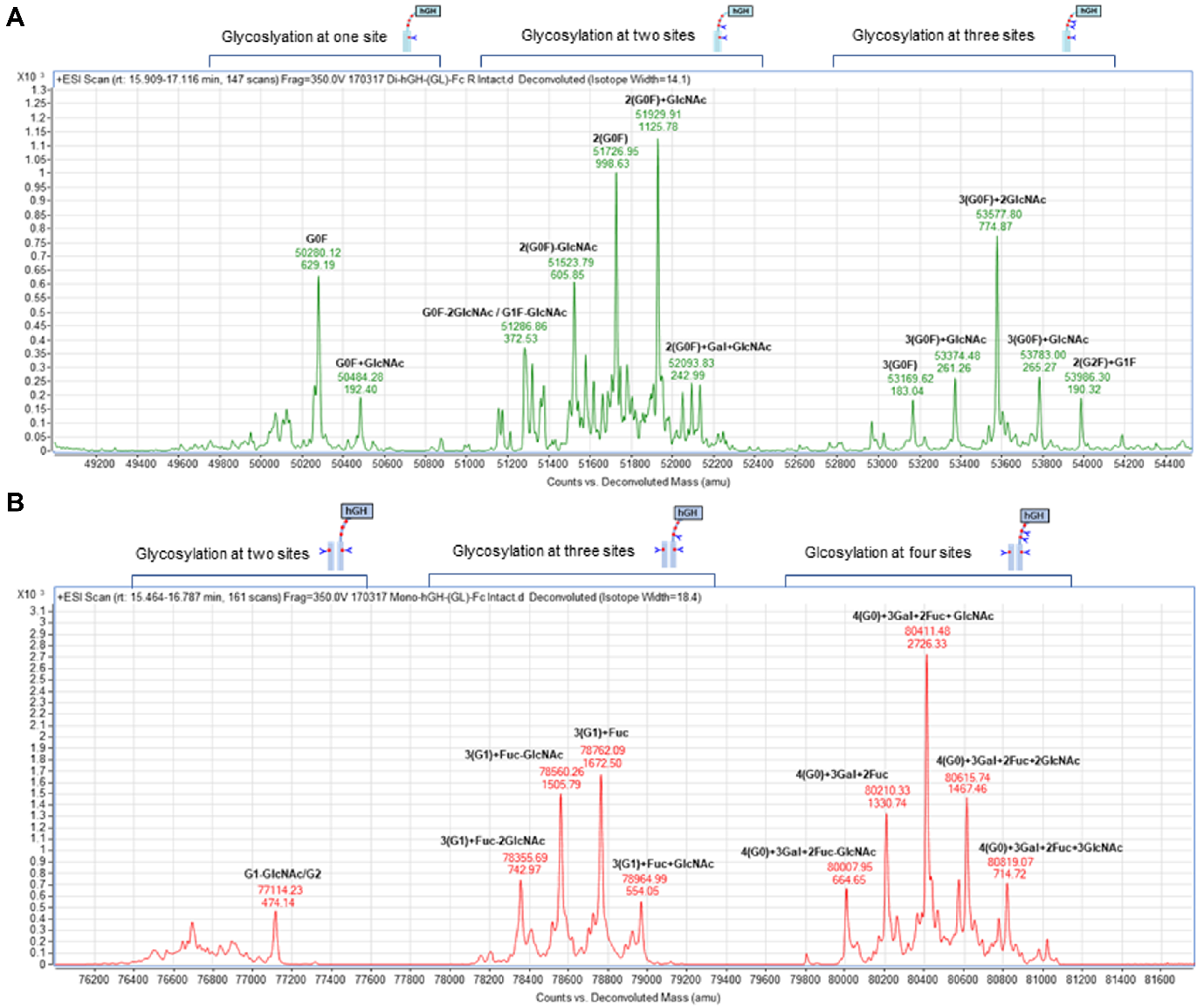


**S2 Fig. Liquid chromatography-mass spectrometry analysis of hGH-Fc fusion protein constructs with a linker containing three engineered N-glycosylation sites.** Human growth hormone (hGH) Fc fusion protein constructs with engineered glycosylation sites in the linker region. Two constructs, Mono-hGH-(GL)-Fc (A) and Di-hGH-(GL)-Fc (B), produced in Chinese hamster ovary cells, were subjected to glycan profiling at the intact protein level. To reduce spectral complexity from oligosaccharide pairs, Di-hGH-(GL) was treated with 50 mM dithiothreitol. Major peaks in the mass spectra were assigned to specific glycan species attached to the protein constructs. Both constructs exhibited heterogeneity in glycoform composition and glycan site occupancy. Notably, no sialylation was observed in the analyzed samples. A schematic representation of the most probable glycosylated construct structures is shown above each mass spectrum for visual reference.
