# Supplementary material for: Engineering of long-acting human growth hormone-Fc fusion proteins: Effects of valency, fusion position, and linker design on pharmacokinetics and efficacy
Source: PLoS One. 2025 May 15;20(5):e0323791. doi: 10.1371/journal.pone.0323791 (PMC12080763; doi:10.1371/journal.pone.0323791)
Supplement: S1 Table — This table provides the construct names, linker sequences, and calculated theoretical masses of reduced monomeric Di-hGH-(GS3)-Fc fusion proteins after dithiothreitol treatment. The control construct contains three GGGGS sequence repeats (GS3 linker), while glycoengineered constructs (Glyc1, Glyc2, and Glyc3) feature an N-glycosylation sequon (NGT) at different positions within the GS3 linker: the first repeat (Glyc1), second repeat (Glyc2), and third repeat (Glyc3). Differences in theoretical mass between the control and glycoengineered constructs reflect the amino acid substitutions required to introduce the N-glycosylation sites. (DOCX) [file pone.0323791.s003.docx]

**S1 Table. Theoretical masses and linker sequences of reduced monomeric Di-hGH-(GS3)-Fc fusion protein constructs containing engineered N-glycosylation sites.**

| Construct name | Linker sequence (GS3) | Theoretical mass (Da) |
| --- | --- | --- |
| Di-hGH-(GS3)-Fc | GGGGS GGGGS GGGGS | 48,455,55 |
| Di-hGH-(GS3)-Fc__Glyc1_ | GGG**NGT** GGGGS GGGGS | 48,583.68 |
| Di-hGH-(GS3)-Fc__Glyc2_ | GGGGS GGG**NGT** GGGGS | 48,583.68 |
| Di-hGH-(GS3)-Fc__Glyc3_ | GGGGS GGGGS GGG**NGT** | 48,583.68 |

**NGT** is the specific amino acid sequence for N-glycosylation; N is asparagine; G is glycine; T is threonine.

This table provides the construct names, linker sequences, and calculated theoretical masses of reduced monomeric Di-hGH-(GS3)-Fc fusion proteins after dithiothreitol treatment. The control construct contains three GGGGS sequence repeats (GS3 linker), while glycoengineered constructs (Glyc1, Glyc2, and Glyc3) feature an N-glycosylation sequon (NGT) at different positions within the GS3 linker: the first repeat (Glyc1), second repeat (Glyc2), and third repeat (Glyc3). Differences in theoretical mass between the control and glycoengineered constructs reflect the amino acid substitutions required to introduce the N-glycosylation sites.
