# Supplementary material for: Engineering of long-acting human growth hormone-Fc fusion proteins: Effects of valency, fusion position, and linker design on pharmacokinetics and efficacy
Source: PLoS One. 2025 May 15;20(5):e0323791. doi: 10.1371/journal.pone.0323791 (PMC12080763; doi:10.1371/journal.pone.0323791)
Supplement: S2 Table — This table presents the theoretical mass data for various glycan species found in hGH-Fc fusion protein constructs with glycosylated linkers. The glycan structures are represented using symbols: ◇ Sialic acid, ○ Galactose, □ GlcNAc, ● Mannose, △ Fucose. The table includes the glycan composition and the corresponding theoretical mass for each glycan species. (DOCX) [file pone.0323791.s004.docx]

**S2 Table. Glycan species of hGH-Fc fusion protein constructs with glycosylated linkers.** Theoretical mass data of glycan species

| Glycan species | Structure | Theoretical mass (Da) |
| --- | --- | --- |
| G0 |  | 1,298.476 |
| G0-GlcNAc |  | 1,095.397 |
| G0F |  | 1,444.534 |
| G0F-GlcNAc |  | 1,241.455 |
| G0F-2GlcNAc |  | 1,038.376 |
| G0F+GlcNAc |  | 1,647.613 |
| G1 |  | 1,460.529 |
| G1F |  | 1,606.587 |
| G1F-GlcNAc |  | 1,403.507 |
| G2F |  | 1,768.640 |

This table presents the theoretical mass data for various glycan species found in hGH-Fc fusion protein constructs with glycosylated linkers. The glycan structures are represented using symbols: ◇ Sialic acid, ○ Galactose, □ GlcNAc, ● Mannose, △ Fucose. The table includes the glycan composition and the corresponding theoretical mass for each glycan species.
