# Supplementary material for: Engineering of long-acting human growth hormone-Fc fusion proteins: Effects of valency, fusion position, and linker design on pharmacokinetics and efficacy
Source: PLoS One. 2025 May 15;20(5):e0323791. doi: 10.1371/journal.pone.0323791 (PMC12080763; doi:10.1371/journal.pone.0323791)
Supplement: S3 Table — This table presents the theoretical masses of non-glycosylated hGH-Fc fusion protein backbones for both monomeric Di-hGH-(GL)-Fc and heterodimeric Mono-hGH-(GL)-Fc forms. (DOCX) [file pone.0323791.s005.docx]

**S3 Table. Glycan species of hGH-Fc fusion protein constructs with glycosylated linkers.** Theoretical mass data of hGH-Fc fusion protein.

| Construct name | Theoretical mass with no glycan (Da) |
| --- | --- |
| Di-hGh-(GL)-Fc | 48,839.94 (monomer) |
| Mono-hGH-(GL)-Fc | 74238.6 (heterodimer) |

This table presents the theoretical masses of non-glycosylated hGH-Fc fusion protein backbones for both monomeric Di-hGH-(GL)-Fc and heterodimeric Mono-hGH-(GL)-Fc forms.
