# Supplementary material for: Engineering of long-acting human growth hormone-Fc fusion proteins: Effects of valency, fusion position, and linker design on pharmacokinetics and efficacy
Source: PLoS One. 2025 May 15;20(5):e0323791. doi: 10.1371/journal.pone.0323791 (PMC12080763; doi:10.1371/journal.pone.0323791)
Supplement: S4 Table — This table lists the theoretical masses of individual monosaccharides that compose the glycan structures found in the fusion proteins. (DOCX) [file pone.0323791.s006.docx]

**S4 Table. Glycan species of hGH-Fc fusion protein constructs with glycosylated linkers.** Theoretical mass data of monosaccharides.

| Monosaccharides | Monoisotonic residue mass (Da) |
| --- | --- |
| N-Acetylglucosamine (GlcNAc) | 203.079 |
| Galactose (Gal) | 162.053 |
| Sialic acid (SA) | 291.095 |
| Mannose (Man) | 180.156 |
| Fucose (Fuc) | 146.058 |

This table lists the theoretical masses of individual monosaccharides that compose the glycan structures found in the fusion proteins.
