# Supplementary material for: Engineering of long-acting human growth hormone-Fc fusion proteins: Effects of valency, fusion position, and linker design on pharmacokinetics and efficacy
Source: PLoS One. 2025 May 15;20(5):e0323791. doi: 10.1371/journal.pone.0323791 (PMC12080763; doi:10.1371/journal.pone.0323791)
Supplement: S5 Table — This table summarizes the glycan species of hGH-Fc fusion protein constructs with glycosylated linkers. Theoretical masses of glycosylated hGH-Fc fusion proteins were calculated using data from S2–S4 Tables to assign major peaks observed in S2 Fig to specific glycan species. These predictions were based on matching experimentally determined peak masses from S2 Fig to the theoretical masses of glycosylated constructs. (DOCX) [file pone.0323791.s007.docx]

**S5 Table. Glycan species of hGH-Fc fusion protein constructs with glycosylated linkers.** Predicted glycan composition of glycosylated hGH-Fc fusion proteins with a linker containing N-linked glycosylation sites

| Construct | Glycan composition | Experimental mass (Da) | Calculated mass (Da) |
| --- | --- | --- | --- |
| Di-hGH-(GL)-Fc | G0F | 50,280.12 | 50,284.47 |
|  | G0F+GlcNAc | 50,484.28 | 50,487.55 |
|  | G0F-2GlcNAc/G1F-GlcNAc | 51,285.86 | 51,281.82 |
|  | 2(G0F)-GlcNAc | 51,523.79 | 51,525.93 |
|  | 2(G0F) | 51,726.95 | 51,729.01 |
|  | 2(G0F)+GlcNAc | 51,929.91 | 51,932.09 |
|  | 2(G0F)+Gal+GlcNAc | 52,093.83 | 52,094.14 |
|  | 3(G0F)-GlcNAc | 52,970.27 | 52,970.46 |
|  | 3(G0F) | 53,169.62 | 53,173.54 |
|  | 3(G0F)+GlcNAc | 53,374.48 | 53,376.62 |
|  | 3(G0F)+2GlcNAc | 53,577.80 | 53,579.70 |
|  | 3(G0F)+3GlcNAc | 53,783.00 | 53,782.78 |
|  | 2(G2F)+G1F | 53,986.30 | 53,983.81 |
| Mono-hGH-(GL)-Fc | G1-GlcNAc/G2 | 77,114.23 | 77,118.63 |
|  | 3(G1)+Fuc-2GlcNAc | 78,355.69 | 78,360.10 |
|  | 3(G1)+Fuc-GlcNAc | 78,560.26 | 78,563.18 |
|  | 3(G1)+Fuc | 78,762.09 | 78,766.25 |
|  | 3(G1)+Fuc+GlcNAc | 78,964.99 | 78,969.33 |
|  | 4(G0)+3Gal+2Fuc-GlcNAc | 80,007.95 | 80,007.72 |
|  | 4(G0)+3Gal+2Fuc | 80,210.33 | 80,210.80 |
|  | 4(G0)+3Gal+2Fuc+ GlcNAc | 80,411.48 | 80,413.88 |
|  | 4(G0)+3Gal+2Fuc+2GlcNAc | 80,615.74 | 80,616.96 |
|  | 4(G0)+3Gal+2Fuc+3GlcNAc | 80,819.07 | 80,820.04 |

This table summarizes the glycan species of hGH-Fc fusion protein constructs with glycosylated linkers. Theoretical masses of glycosylated hGH-Fc fusion proteins were calculated using data from S2–S4 Tables to assign major peaks observed in S2 Fig to specific glycan species. These predictions were based on matching experimentally determined peak masses from S2 Fig to the theoretical masses of glycosylated constructs.
