# Supplementary material for: Engineering of long-acting human growth hormone-Fc fusion proteins: Effects of valency, fusion position, and linker design on pharmacokinetics and efficacy
Source: PLoS One. 2025 May 15;20(5):e0323791. doi: 10.1371/journal.pone.0323791 (PMC12080763; doi:10.1371/journal.pone.0323791)
Supplement: S1 raw image — (PDF) [file pone.0323791.s008.pdf]

Fig 2A original blot

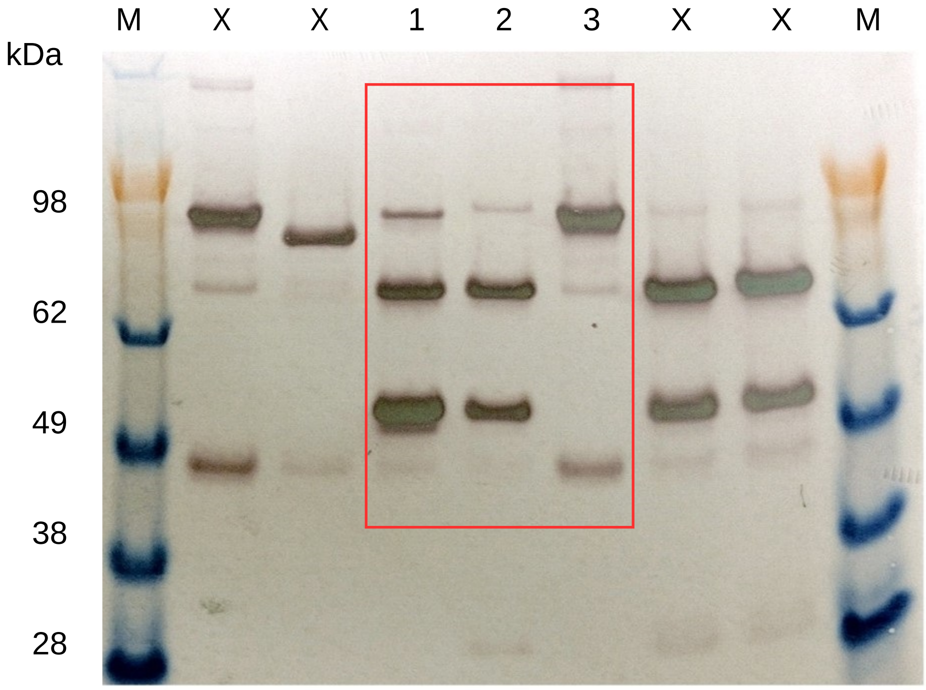

M: SeeBlue® Plus2 Pre-stained Protein Standard
Lane 1: Monovalent hGH-Fc with wild type CH3 Fc domain (Mono-hGH-(FL3)-Fc)
Lane 2: Monovalent hGH-Fc with knob-in-hole (KiH)mutation in the CH3 Fc domains (Mono-hGH-(FL3)-Fc_KiH)
Lane 3: Bivalent hGH-Fc (Di-hGH-(FL3)-Fc)
Image capture method: Fujifilm FinePix S3 Pro)
Red box: indicating regions used in figure

Fig 2B original blot

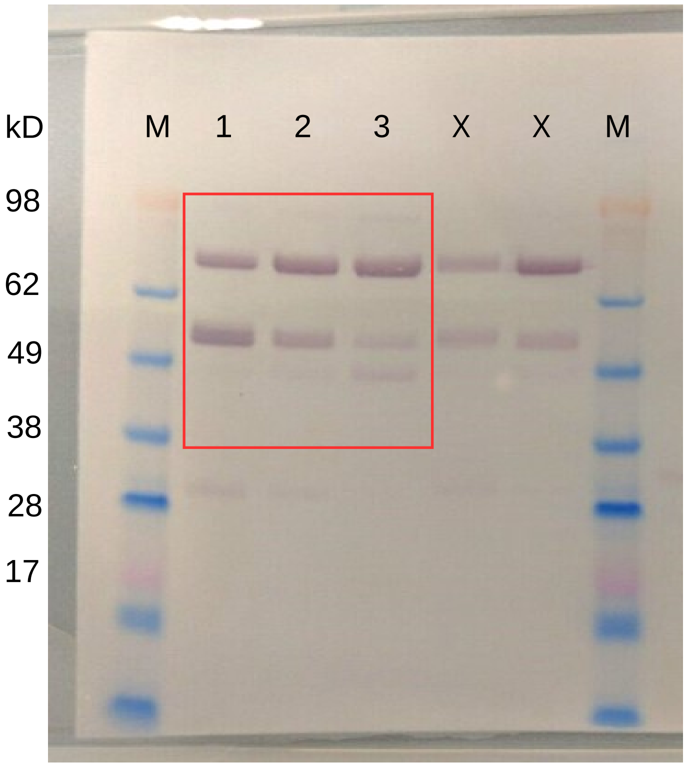

M: SeeBlue Plus2 Pre-stined Protein standard
Lane 1: Mono-hGH-(FL3)-Fc_KiH, single vector (vector 1 expresing hGH-Fc and Fc chain)
Lane 2: Mono-hGH-(FL3)-Fc_KiH, two vectors (vector 1 expressing hGH-Fc+Fc, vector 2 expressing only hGH-Fc), vector ratio of 1 and 2 (1:1)
Lane 3: Mono-hGH-(FL3)-Fc_KiH, two vectors (vector 1 expressing hGH-Fc+Fc, vector 2 expressing only hGH-Fc), vector ratio of 1 and 2 (3:7)
Image capture method: Digital camera (Fujifilm FinePix S3 Pro)
Red box: indicating regions used in figure

Fig 2 C original SDS-PAGE non-reduced gel

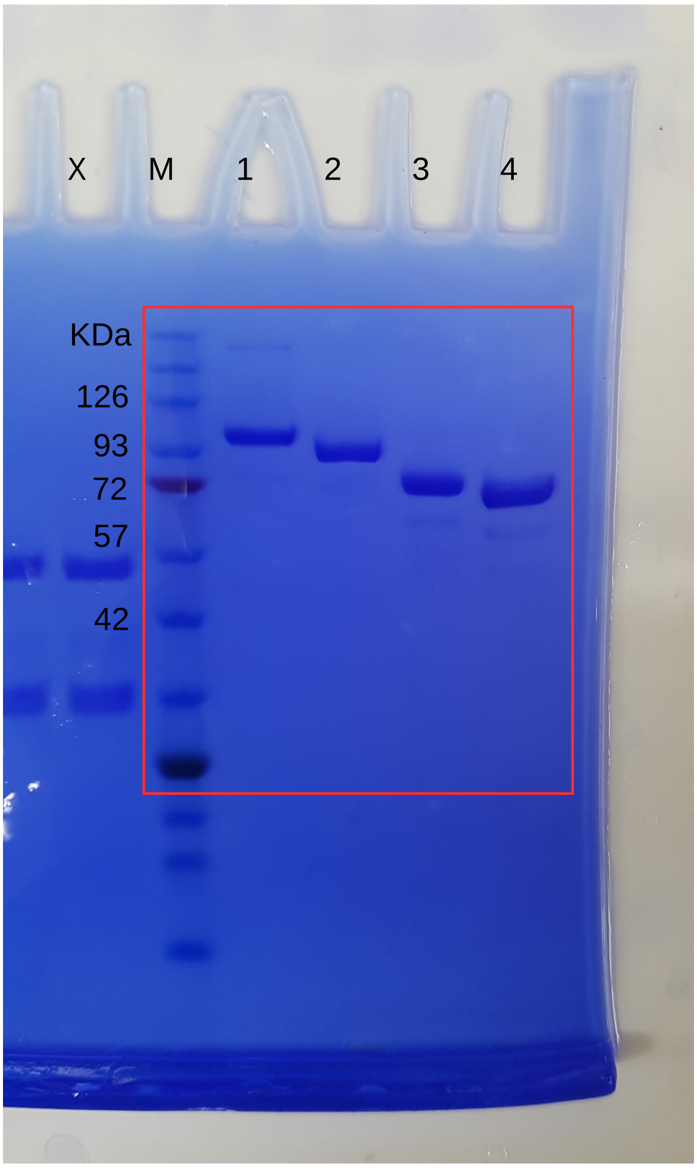

M: Blueye Prestained Protein Ladder
Lane 1: Bivalent hGH-Fc (Di-hGH-(FL3)-Fc)
Lane 2: Bivalent Fc-hGH-Fc (Fc-(FL3)-di-hGH)
Lane 3: Monovalent hGH-Fc (Mono-hGH-(FL3)-Fc)
Lane 4: Monovalent Fc-hGH (Fc-(FL3)-mono-hGH)
Image capture method: Digital camera (Fujifilm FinePix S3 Pro)
Red box: indicating regions used in figure

Fig 2C original SDS-PAGE reduced gel

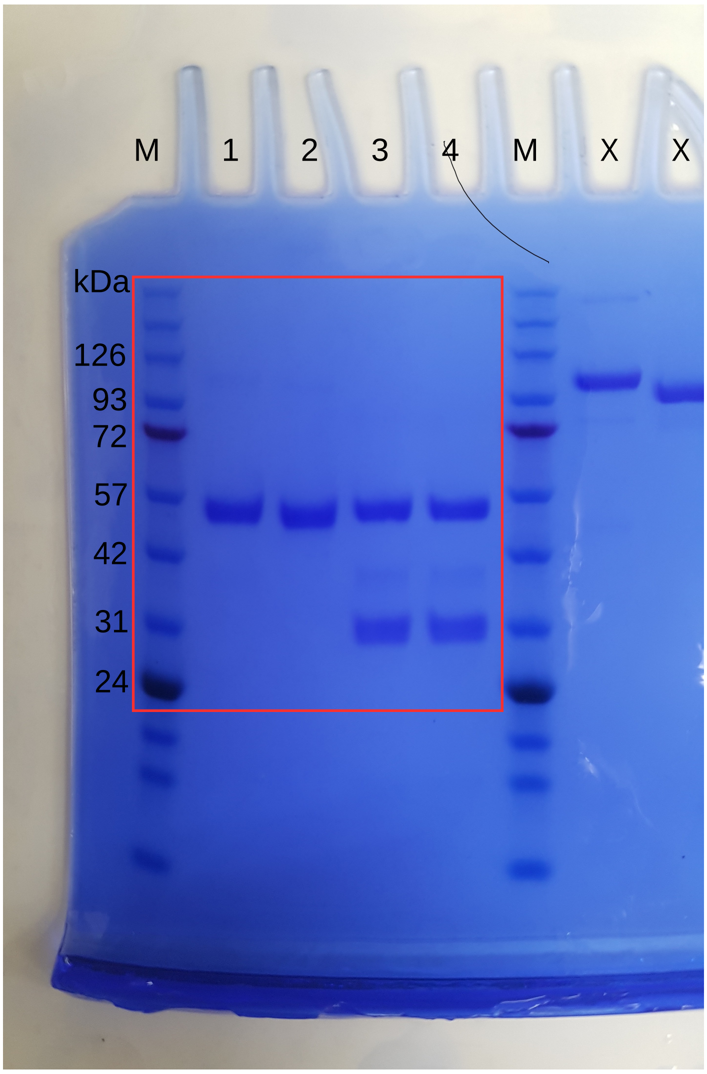

M: Blueye Prestained Protein Ladder
Lane 1: Bivalent hGH-Fc (Di-hGH-(FL3)-Fc)
Lane 2: Bivalent Fc-hGH (Fc-(FL3)-di-hGH)
Lane 3: Monovalent hGH-Fc (Mono-hGH-(FL3)-Fc)
Lane 4: Monovalent Fc-hGH (Fc-(FL3)-mono-hGH)
Image capture method: Digital camera (Fujifilm FinePix S3 Pro)
Red box: indicating regions used in figure
